# Ginsenoside Rg1 Prevents and Treats Acute Pulmonary Injury Induced by High-Altitude Hypoxia

**DOI:** 10.3390/ijms252212051

**Published:** 2024-11-09

**Authors:** Junru Chen, Zhuo Zhang, Mingyue Huang, Jiayi Yan, Rong Gao, Jialu Cui, Yue Gao, Zengchun Ma

**Affiliations:** 1 School of Pharmacy, Guangdong Pharmaceutical University, Guangzhou 510006, China; 15088094928@163.com (J.C.); gaoronger198@163.com (R.G.); 2 Department of Pharmaceutical Sciences, Beijing Institute of Radiation Medicine, Beijing 100850, China; zhangzhuo000@yeah.net (Z.Z.); 15907489303@163.com (M.H.); yjy19951025@163.com (J.Y.); cuijialu143021@163.com (J.C.); 3 Institute of Traditional Chinese Medicine, Tianjin University of Traditional Chinese Medicine, Tianjin 301617, China

**Keywords:** ginsenoside Rg1, high-altitude hypoxia, acute lung injury, fluid shear stress, mitochondrial function, inflammation

## Abstract

This study aimed to investigate the protective effects of ginsenoside Rg1 on high-altitude hypoxia-induced acute lung injury (ALI) and elucidated its molecular targets and related pathways, specifically its association with the fluid shear stress pathway. Using a combination of bioinformatics analysis and both in vivo and in vitro experiments, we assessed the role of ginsenoside Rg1 in mitigating physiological and biochemical disturbances induced by hypoxia. In the in vivo experiments, we measured arterial blood gas parameters, levels of inflammatory cells and cytokines, erythrocyte and platelet parameters, and conducted histological analysis in rats. The in vitro experiments utilized human pulmonary microvascular endothelial cells (HPMECs) and A549 cells to examine cell viability, intracellular reactive oxygen species (ROS) and Ca^2^⁺ levels, and mitochondrial function. The results of the in vivo experiments demonstrate that ginsenoside Rg1 significantly increased arterial blood oxygen partial pressure and saturation, elevated arterial blood glucose levels, and stabilized respiratory and metabolic functions in rats. It also reduced inflammatory cells and cytokines, such as tumor necrosis factor-α and interleukin-6, and improved erythrocyte and platelet abnormalities, supporting its protective role through the regulation of the fluid shear stress pathway. Histological and ultrastructural analyses revealed that Rg1 significantly protected lung tissue structure and organelles. In vitro experiments further confirmed that Rg1 improved cell viability in HPMEC and A549 cells under hypoxic conditions, decreased intracellular ROS and Ca^2^⁺ levels, and enhanced mitochondrial function. These findings collectively demonstrate that ginsenoside Rg1 exerts significant protective effects against high-altitude hypoxia-induced ALI by enhancing oxygen delivery and utilization, reducing inflammatory responses, and maintaining cellular metabolism and vascular function. Notably, the protective effects of Rg1 are closely associated with the regulation of the fluid shear stress pathway, suggesting its potential for treating high-altitude hypoxia-related diseases.

## 1. Introduction

High-altitude regions attract millions of visitors annually for a variety of activities, such as sightseeing, hiking, mountaineering, skiing, and seeking respite from the summer heat. In 2014, Lhasa, Tibet, located at an elevation of 3658 m, drew in more than 15 million tourists [1]. Acute mountain sickness (AMS) is a negative physiological reaction that occurs when individuals are rapidly exposed to altitudes above 2500 m. The respiratory system is particularly susceptible to altitude-related harm, and this can manifest as high-altitude acute lung injury (ALI), which often presents symptoms such as chest tightness, breathlessness, headaches, and difficulty sleeping. In severe cases, this condition can advance to high-altitude pulmonary edema (HAPE). A comprehensive study conducted on a large prospective cohort revealed that the occurrence of HAPE among individuals residing at elevations surpassing 4000 m stands at 1.7% [2]. The implementation of gradual ascent and/or pre-acclimatization methods has proven to be effective in preventing HAPE at high altitudes. This conclusion is supported by data demonstrating that individuals who ascend to 4500 m within a four-day period have a significantly reduced incidence of HAPE, standing at only 0.2%. In contrast, among the same group of individuals who reach this altitude within 1–2 days, the incidence of HAPE increases to 6% [3]. Nevertheless, despite its potential for prevention, HAPE remains a significant cause of morbidity and mortality, partly due to factors such as lack of awareness [4]. As altitude increases and atmospheric pressure decreases, there is a corresponding decline in the partial pressure of oxygen. In response to this hypoxemia, the body initiates various physiological and cellular responses aimed at ensuring sufficient tissue oxygenation. These responses encompass heightened ventilation, heart rate, and cardiac output; cerebrovascular dilation and augmented blood flow; activation of hypoxia-inducible factors (HIFs); as well as the generation of reactive oxygen species (ROS) [5]. ALI is intricately intertwined with these physiological responses, as its mechanisms involve a complex interplay of various physiological and pathological processes. These processes encompass uneven pulmonary vasoconstriction, elevation of pulmonary arterial pressure induced by hypoxia, increased capillary pressure due to over-perfusion, and impaired alveolar fluid clearance. The most effective emergency interventions for ALI include supplemental oxygen and prompt descent to lower altitudes. In terms of pharmacological treatments, acetazolamide, dexamethasone, calcium channel blockers like nifedipine, phosphodiesterase type 5 (PDE5) inhibitors, and β-adrenergic agonists have all been utilized. However, each treatment option carries certain limitations and side effects [6].

Low pressure and hypoxia can induce inflammatory responses, which in turn result in elevated levels of pro-inflammatory cytokines and inflammatory markers, thereby exacerbating the progression of the disease. The interplay between oxidative stress and inflammatory responses reinforces each other, thereby further aggravating ALI [7]. Therefore, antioxidant and anti-inflammatory therapies are considered crucial strategies in the prevention and management of ALI. Traditional Chinese medicine (TCM), with its multi-component, multi-target, and multi-pathway mechanisms, is particularly well suited to addressing the intricate multi-system dysfunctions induced by low pressure and hypoxia. TCM has a long history of use in humans, is known for its clinical safety and lower toxicity, and is often combined with conventional drugs to achieve synergistic effects. In the context of AMS, the primary TCM syndromes manifest as Qi deficiency and blood stasis. The main therapeutic principles involve promoting Qi and blood circulation and resolving phlegm. Commonly used herbal medicines include *Astragalus mongholicus Bunge*, *Salvia miltiorrhiza Bunge*, *Panax ginseng C.A. Mey.*, and *Rhodiola rosea* L., which can help mitigate the effects of hypoxia and support the body’s adaptation to high altitudes [8].

Preclinical and clinical evidence has confirmed the therapeutic effects of ginseng on central nervous system diseases, metabolic disorders, and tumors, primarily due to its anti-inflammatory and antioxidant properties. Ginsenoside Rg1, identified as a quality control component of ginseng in the 2020 edition of the *Chinese Pharmacopoeia*, exhibits multi-target pharmacological activities across various organs and tissues, including the brain and liver [9]. Research has demonstrated that Rg1 can improve hypoxic pulmonary hypertension (HPH). This effect is possibly mediated through the regulation of cellular communication network factor 1 (CCN1) expression [10] or by acting on the phosphoinositide 3-kinase (PI3K)/protein kinase B (AKT) signaling pathway [11]. However, the potential mechanisms by which Rg1 improves hypoxia-induced ALI have yet to be fully elucidated. In this study, we employed network pharmacology to determine that Rg1 may prevent and treat high-altitude hypoxia-induced ALI through the shear stress pathway. Shear stress is the frictional force exerted by blood flow on the vascular endothelium. It plays a crucial role in maintaining the structure and function of endothelial cells (ECs) and is a key hemodynamic force in sustaining endothelial homeostasis and vascular health. Shear stress has numerous beneficial effects, including anti-inflammatory, anti-proliferative, anti-apoptotic, anti-monocyte adhesion, and anti-thrombotic actions [12]. ECs’ mitochondria are not only the main source of ROS but also the targets of ROS-induced damage. They play a critical role in maintaining cytoplasmic Ca^2+^ concentration. Disruption of mitochondrial homeostasis can trigger inflammation and stimulate immune signaling cascades, leading to endothelial dysfunction. Recently, increasing evidence has shown that laminar shear stress (LSS) increases the mitochondrial inner membrane potential, promotes mitochondrial fusion, and enhances the expression of antioxidant enzymes [13,14]. In contrast, oscillatory shear stress (OSS) promotes the production of mitochondrial superoxide, which can induce apoptosis [15]. This distinction between the effects of LSS and OSS underscores the importance of the nature of shear stress in vascular health and disease. Our results indicated that ginsenoside Rg1 could improve blood viscosity in rats following hypoxia and reduce ROS release and Ca^2+^ influx in human pulmonary microvascular endothelial cells (HPMECs) and A549 cells (a commonly used human lung adenocarcinoma cell line). This suggests that Rg1 is a potential therapeutic agent for the prevention and treatment of ALI. In this study, our aim was to investigate the effects of ginsenoside Rg1 on blood viscosity and cellular oxidative stress under hypoxic conditions, and to evaluate its potential as a therapeutic intervention for ALI.

## 2. Results

### 2.1. Rg1 Target-Pathway and High Altitude-Induced ALI Target Network Analysis

**Figure 1:**
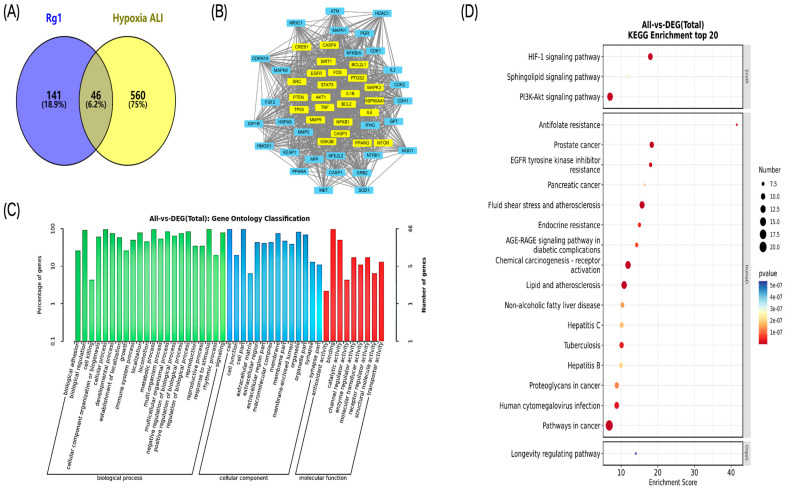
Pharmacological analysis of Rg1 targets and high altitude-induced ALI: PPI network and hub genes. (**A**) Venn diagram showing the overlap between Rg1 targets and targets associated with ALI induced by high-altitude hypoxia; (**B**) PPI network topology analysis of the intersection genes, with the core targets highlighted in yellow; (**C**) bar graph illustrating the GO enrichment analysis of the intersection genes; (**D**) bubble plot displaying the KEGG enrichment analysis of the intersection genes.

A comprehensive analysis identified 187 potential effective targets of Rg1. By integrating and deduplicating data from five databases, 606 disease-related genes associated with high altitude-induced ALI were obtained. The intersection of these datasets revealed 46 common targets (Figure 1A). Key targets within the PPI network included TNF, IL6, STAT3, AKT1, and TP53 (Figure 1B). Enriched biological processes (BPs) encompassed biological adhesion and regulation, while enriched molecular functions (MFs) included antioxidant activity and channel regulator activity. Enriched cellular components (CCs) comprised cell junctions and cellular components (Figure 1C). Figure 1D demonstrated that the core pathways through which Rg1 may exert protective effects against high altitude-induced ALI include the fluid shear stress and atherosclerosis pathway, the HIF-1 signaling pathway, and the PI3K-AKT signaling pathway.

### 2.2. Rg1 Alleviated the Physiological Stress Response Induced by High-Altitude Hypoxia

**Figure 2:**
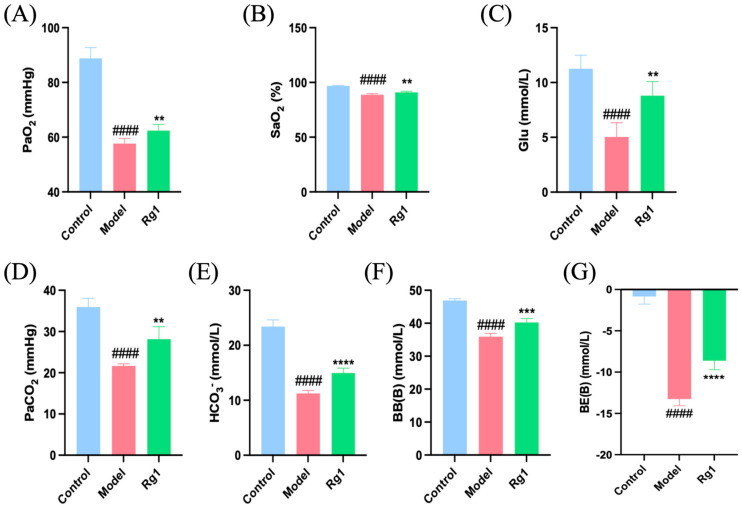
Blood gas analyses results of abdominal aorta blood in rats from each group. (**A**) PaO_2_; (**B**) SpO_2_; (**C**) glucose concentration; (**D**) partial pressure of arterial carbon dioxide (PaCO_2_); (**E**) HCO_3_^−^ concentration; (**F**) BB concentration; (**G**) BE concentration. ** *p* < 0.01, *** *p* < 0.001, **** *p* < 0.0001 versus the Model group; ^####^ *p* < 0.0001 versus the Control group (*n* = 5, $\bar{x}$ ± s).

Figure 2A,B demonstrated that Rg1 effectively increased the partial pressure of arterial oxygen (PaO_2_) and blood oxygen saturation (SpO_2_) in rats, thereby alleviating hypoxia. Additionally, Rg1 significantly elevated glucose levels in arterial blood (Figure 2C), suggesting an enhanced energy supply to better cope with hypoxic conditions. In a low-pressure, low-oxygen environment, rats exhibited respiratory alkalosis due to hyperventilation and excessive CO_2_ excretion, resulting in arterial blood CO_2_ partial pressure falling below normal levels. As a compensatory response, the concentrations of bicarbonate (HCO_3_^−^) and buffer base (BB) decreased, while the base excess (BE) value increased. Following Rg1 administration, improvements in the respiratory alkalosis condition were observed (Figure 2D–G).

### 2.3. Rg1 Alleviated the Inflammatory Response Induced by High-Altitude Hypoxia

**Figure 3:**
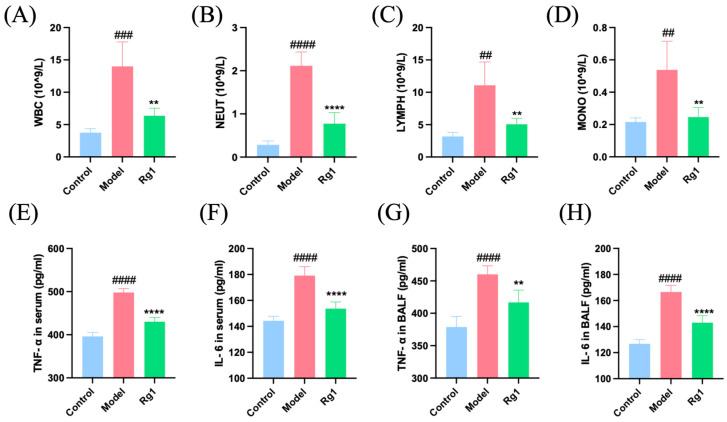
Measurement of inflammatory cells and inflammatory factors in rats from each group. (**A**) WBCs; (**B**) NEUTs; (**C**) LYMPHs; (**D**) MONOs in the abdominal aorta blood; (**E**) TNF-α concentration in serum; (**F**) IL-6 concentration in serum from the abdominal aorta blood; (**G**) TNF-α concentration in BALF; (**H**) IL-6 concentration in BALF. ** *p* < 0.01, **** *p* < 0.0001 versus the Model group; ^##^ *p* < 0.01, ^###^ *p* < 0.001, ^####^ *p* < 0.0001 versus the Control group (*n* = 5, $\bar{x}$ ± s).

Compared to the Control group, the Model group of rats exhibited a significant increase in the number of WBCs, neutrophils (NEUTs), lymphocytes (LYMPHs), and monocytes (MONOs) in the blood of the abdominal aorta. Following the administration of Rg1, these levels were significantly reduced. Additionally, the levels of TNFα and IL-6 in the serum and alveolar lavage fluid of the model group were elevated but decreased after Rg1 treatment (Figure 3).

### 2.4. Rg1 Decreased Blood Viscosity in Hypoxic Rats

**Figure 4:**
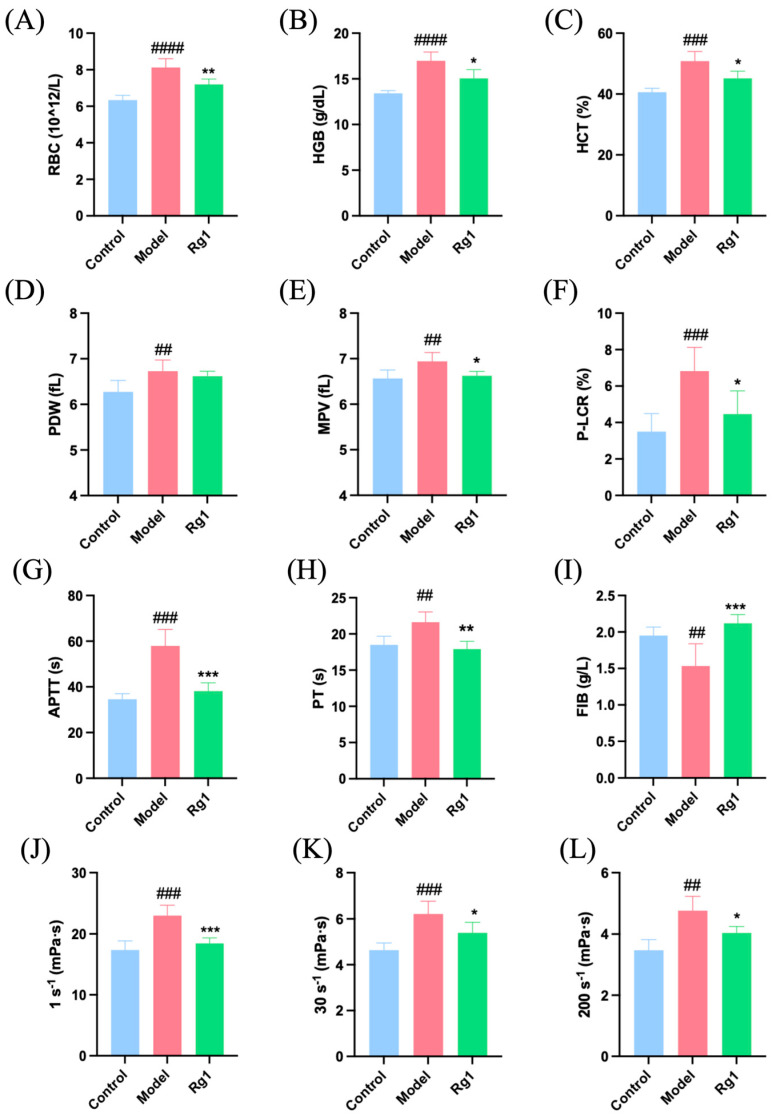
Blood viscosity measurements in rats from each group. (**A**) RBCs; (**B**) HGB; (**C**) HCT; (**D**) platelet distribution width (PDW); (**E**) mean platelet volume (MPV); (**F**) platelet–larger cell ratio (P-LCR); (**G**) APTT; (**H**) PT; (**I**) FIB levels in the abdominal aorta blood; (**J**) low-shear whole blood viscosity (mPas); (**K**) medium-shear whole blood viscosity (mPas); (**L**) high-shear whole blood viscosity (mPas). * *p* < 0.05, ** *p* < 0.01, *** *p* < 0.001 versus the Model group; ^##^ *p* < 0.01, ^###^ *p* < 0.001, ^####^ *p* < 0.0001 versus the Control group (*n* = 5, $\bar{x}$ ± s).

Under hypoxic conditions, rats exhibited compensatory increases in RBCs, leading to elevated levels of HGB and hematocrit (HCT). Additionally, the platelet size distribution became uneven, with increased distribution width and mean volume, as well as a higher proportion of large platelets. After the administration of Rg1, these indices showed improvement (Figure 4A–F). Furthermore, coagulation tests revealed that Rg1 effectively ameliorated coagulation abnormalities induced by hypoxia (Figure 4G–I). Hemorheological tests indicated that hypoxic rats had higher low, medium, and high shear viscosity values, suggesting high levels of RBC aggregation and poor deformability. Rg1 administration resulted in a decrease in blood viscosity following hypoxia (Figure 4J–L).

### 2.5. Rg1 Reduced the Pathological Damage of Lung Tissue in Hypoxic Rats

**Figure 5:**
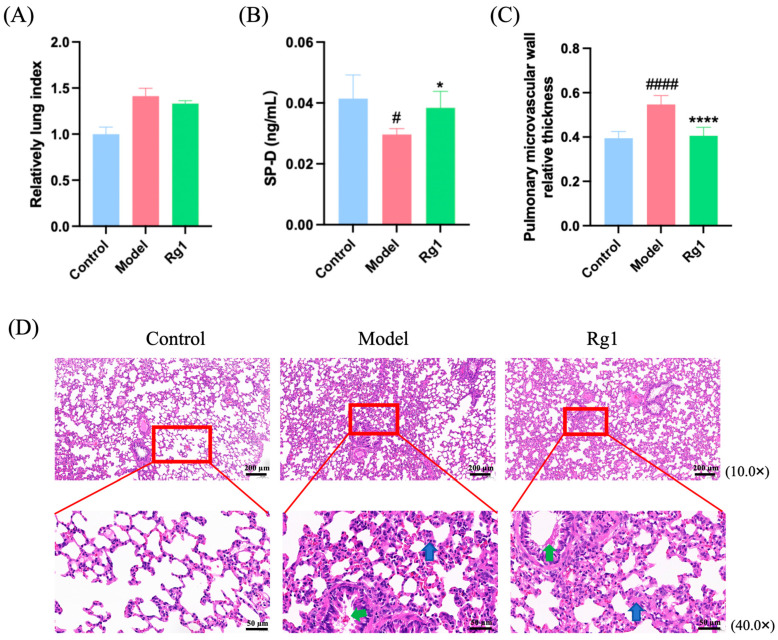
Lung injury assessment in rats from each group. (**A**) Relative lung index of rats, which was calculated as the lung index of each rat/lung index of the Control group rat; (**B**) SP-D content in lung tissue of rats in each group; (**C**) pulmonary microvascular wall relative thickness, which was calculated as the ratio of the wall thickness to the microvascular radius. * *p* < 0.05, **** *p* < 0.001 versus the Model group; ^#^ *p* < 0.05, ^####^ *p* < 0.0001 versus the Control group (*n* = 5, $\bar{x}$ ± s). (**D**) H and E staining results of rat lung tissue (*n* = 3). Following exposure to hypoxic conditions, structural abnormalities were observed, characterized by significantly thickened alveolar walls in numerous regions, as indicated by the blue arrows. Additionally, desquamation of bronchial epithelial cells was noted, with detached cells and proteinaceous mucus evident within the lumens, as denoted by the green arrows.

As demonstrated in Figure 5A, the lung index of hypoxic rats was elevated compared to the control group; however, it decreased following Rg1 administration. Furthermore, Rg1 effectively reduced levels of SP-D protein in lung tissue, as shown in Figure 5B. SP-D is an innate immune protein that plays a crucial role in pulmonary immune defense and inflammatory response and is also involved in immune and inflammatory responses in other organs [16]. It was evident that the pulmonary microvascular walls in the model rats were significantly thickened, and this condition improved following Rg1 administration (Figure 5C). H and E staining of lung tissues from each group revealed that the control group exhibited essentially normal lung tissue structure, characterized by well-defined alveolar contours, intact architecture, thin alveolar walls, and a clear alveolar epithelial cell structure; notably, there was no significant infiltration of inflammatory cells. In contrast, exposure to hypoxia resulted in structural abnormalities, including markedly thickened alveolar walls, as indicated by the blue arrows. Additionally, bronchial epithelial cells were observed to undergo desquamation, with detached cells and proteinaceous mucus present within the lumens, as indicated by the green arrows. After Rg1 administration, the extent of lung injury in rats was significantly ameliorated (Figure 5D).

### 2.6. Rg1 Ameliorated the Structural Abnormalities of Alveolar Epithelial Cells in Hypoxic Rats

**Figure 6:**
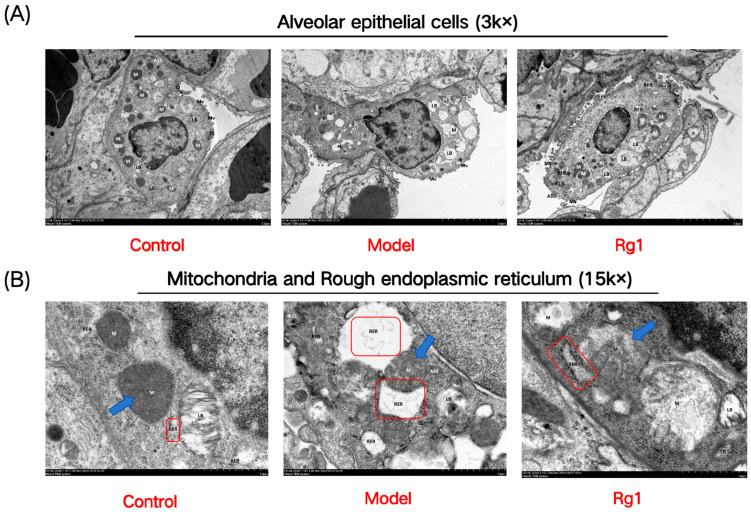
Morphological and subcellular structure observations of alveolar epithelial cells in rats from each group. (**A**) Morphology of alveolar epithelial cells as observed under transmission electron microscopy; (**B**) Mitochondrial structure in alveolar epithelial cells (indicated by blue arrow); rough endoplasmic reticulum in alveolar epithelial cells (indicated by red box) (*n* = 3).

Rg1 treatment resulted in cells exhibiting only mild damage, with irregular shapes but clear boundaries, uniform cytoplasm, and abundant organelles (Figure 6A). Within the control group, mitochondria were round or short and rod-shaped, displaying a normal architecture with intact membranes, uniform matrices, and parallel cristae. Post-hypoxia, mitochondria exhibited significant swelling, with fractured or absent cristae. In the Rg1-treated group, mitochondria showed only mild swelling, with mostly intact outer membranes, a lightened intramembrane matrix, and a few remaining cristae (Figure 6B). Regarding the rough endoplasmic reticulum, control group observations revealed a normal appearance with visible ribosome attachment on the surface. Hypoxic conditions led to membrane damage, ribosome detachment, and vacuolization. In the Rg1 group, there was slight expansion in certain areas, accompanied by some ribosome detachment (Figure 6B).

### 2.7. Establishment of a Cell Hypoxia Model and Preliminary Detection of Rg1 Efficacy

**Figure 7:**
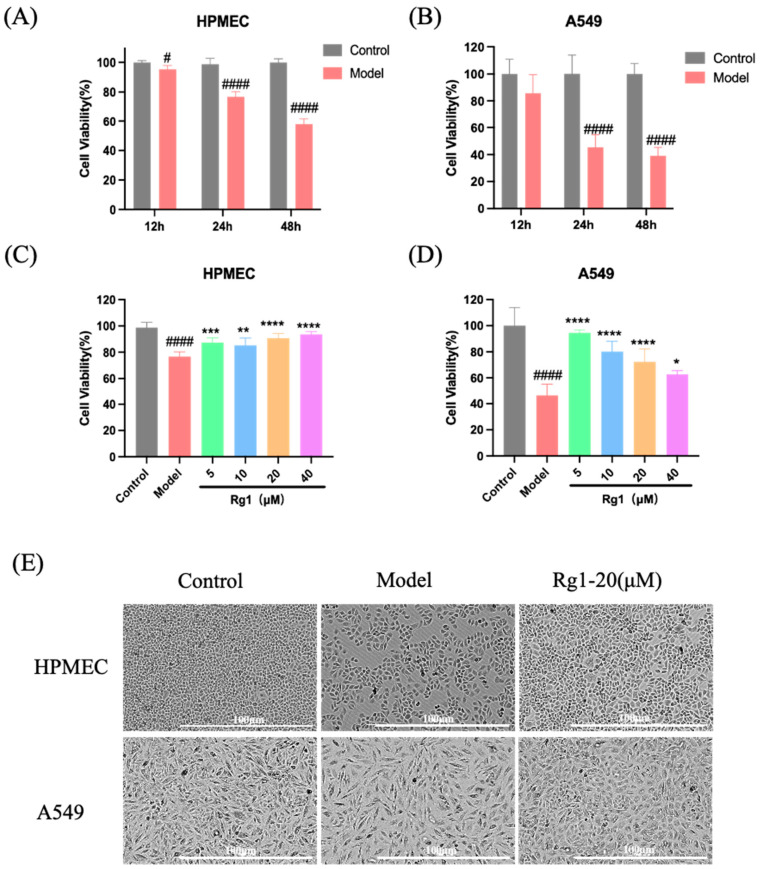
Construction of cell hypoxia model and preliminary verification of Rg1 efficacy. (**A**) Viability of HPMEC after 12, 24, and 48 h of hypoxia under 1% O_2_ and 5% CO_2_ conditions; (**B**) viability of A549 cells after the same hypoxic conditions. After adding 0, 5, 10, 20, and 40 µM Rg1 and implementing 24 h of hypoxia under 1% O_2_ and 5% CO_2_ conditions: (**C**) viability of HPMEC; (**D**) viability of A549 cells; (**E**) Bright-field images of HPMEC and A549 cells after 24 h of hypoxia with 20 µM Rg1 under 1% O_2_ and 5% CO_2_ conditions. * *p* < 0.05, ** *p* < 0.01, *** *p* < 0.001, **** *p* < 0.0001 versus the Model group; ^#^ *p* < 0.05, ^####^ *p* < 0.0001 versus the Control group (*n* = 6, $\bar{x}$ ± s).

HPMEC and A549 cells were exposed to hypoxic conditions with 1% O_2_ and 5% CO_2_ for durations of 12, 24, and 48 h. Cell viability was measured using the CCK8 assay, which indicated a significant reduction in viability after 24 h of hypoxia. Consequently, 24 h was selected as the optimal duration for hypoxic treatment (Figure 7A,B). Subsequently, cells were treated with Rg1 at concentrations of 0, 5, 10, 20, and 40 µM and subjected to 24 h of hypoxia under identical conditions. The findings demonstrated a marked decrease in cell viability under hypoxia; however, Rg1 at various concentrations notably enhanced the survival rates of both cell types (Figure 7C,D). Bright-field microscopy further revealed that 20 µM Rg1 increased cell density under hypoxic conditions (Figure 7E).

### 2.8. Rg1 Reduced ROS Release and Ca^2+^ Influx in Hypoxic Cells

**Figure 8:**
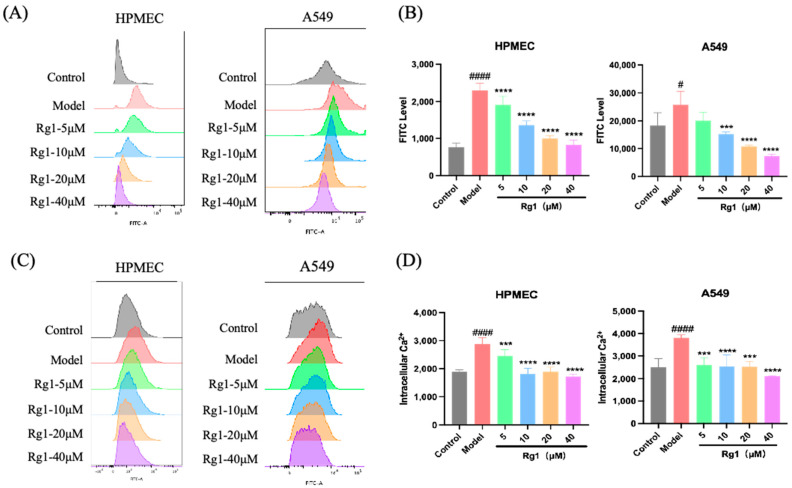
Detection of ROS release and Ca^2+^ influx in cells under hypoxic conditions. After treatment with 0, 5, 10, 20, and 40 µM Rg1 and 24 h of hypoxia under 1% O_2_ and 5% CO_2_ conditions, flow cytometry was used to assess the following in HPMEC and A549 cells: (**A**) Representative images of ROS release: illustrated the effect of different concentrations of Rg1 on ROS levels; (**B**) Statistical graph of ROS release: quantified the ROS release across different Rg1 concentrations; (**C**) Representative images of Ca^2+^ influx: showed the impact of Rg1 on calcium influx in hypoxic cells; (**D**) Statistical graph of Ca^2+^ influx: provided a quantitative analysis of calcium influx at various Rg1 concentrations. *** *p* < 0.001, **** *p* < 0.0001 versus the Model group; ^#^ *p* < 0.05 ^####^ *p* < 0.0001 versus the Control group (*n* = 6, $\bar{x}$ ± s).

Under hypoxic conditions, there was a marked increase in ROS levels in HPMEC and A549 cells, indicating the presence of oxidative stress. Excessive ROS can cause cellular damage, including lipid peroxidation, protein oxidation, and DNA damage, which may be a critical mechanism through which hypoxia induces cell dysfunction and death [17] (Figure 8A,B). Moreover, there was a notable rise in intracellular Ca^2+^ levels, suggesting an imbalance in Ca^2+^ influx. Calcium is a vital intracellular second messenger, and in abnormal concentrations, it can initiate various physiological and pathological responses, such as the activation of proteases, phospholipases, and nucleases, ultimately leading to cellular damage and apoptosis [18] (Figure 8C,D). HPMEC and A549 cells may undergo severe cellular stress and damage due to oxidative stress and disrupted calcium influx. Treatment with Rg1 at concentrations of 5, 10, 20, and 40 μM for 24 h significantly ameliorated these conditions, with the 40 μM concentration exhibiting the most pronounced effect.

### 2.9. Rg1 Improved Mitochondrial Respiration Under Hypoxic Conditions

**Figure 9:**
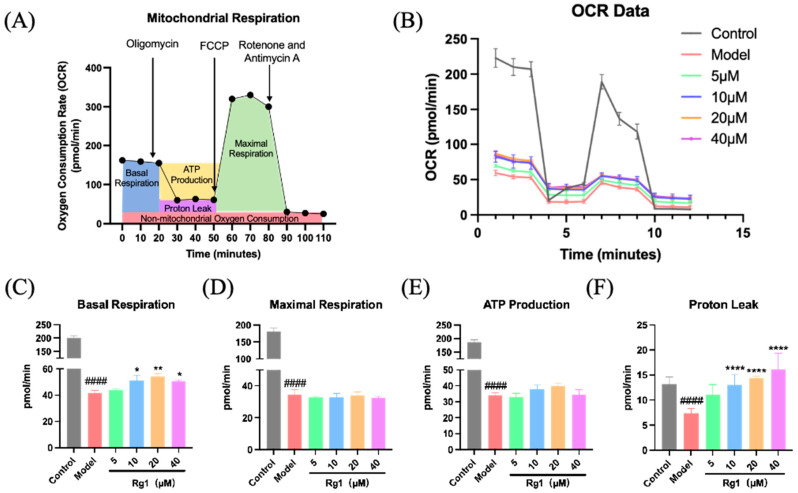
Detection of mitochondrial respiration in HPMEC under hypoxic conditions. After treatment with 0, 5, 10, 20, and 40 µM Rg1 and 24 h of hypoxia under 1% O_2_ and 5% CO_2_ conditions, the mitochondrial respiration of HPMECs was assessed using the Seahorse XFe96 Analyzer: (**A**) Respiration rate schematic illustrating the overall respiration activity of the cells under different Rg1 concentrations; (**B**) OCR: showed the impact of Rg1 on the overall oxygen consumption by the cells; (**C**) Basal respiration: represented the basic energy demand of the cells under hypoxic conditions and the effect of Rg1; (**D**) Maximal respiration: indicated the maximum respiratory capacity of the cells in the presence of Rg1; (**E**) ATP production: demonstrated how Rg1 influenced ATP generation in hypoxic cells; (**F**) Proton leak: displayed the effect of Rg1 on proton leakage, which could be indicative of mitochondrial efficiency and integrity. * *p* < 0.05, ** *p* < 0.01, **** *p* < 0.0001 versus the Model group; ^####^ *p* < 0.0001 versus the Control group (*n* = 6, $\bar{x}$ ± s).

Under hypoxic conditions, the mitochondrial function of HPMEC was significantly compromised, as demonstrated by reductions in several key indicators, including oxygen consumption rate (OCR), basal respiration, maximal respiration, ATP production, and proton leak. Treatment with Rg1 enhanced mitochondrial function in HPMEC, notably by improving basal respiration and reducing proton leakage (Figure 9C,F).

## 3. Discussion

Due to the reduced atmospheric oxygen partial pressure at high altitudes, the resulting hypoxic environment leads to an insufficient supply of oxygen to tissues and organs. This often triggers a series of physiological and pathological responses, including oxidative stress, inflammatory reactions, and cellular damage. Current treatment methods primarily focus on supportive care and symptomatic relief, but their efficacy is limited, and they often have numerous side effects. Therefore, the identification of effective natural compounds for the prevention and treatment of altitude-induced ALI is of great significance. Ginseng is well known for its properties of invigorating vital energy, restoring pulse, and alleviating stagnation. It contains various saponins, among which ginsenoside Rg1 is a principal active component. Extensive research has demonstrated that Rg1 exhibits numerous biological functions, including antioxidative, anti-inflammatory, and cytoprotective effects [19]. However, the precise mechanisms by which Rg1 mitigates ALI induced by high-altitude hypoxia remain poorly understood. Thus, this study aimed to elucidate the protective effects of Rg1 on ALI under high-altitude hypoxic conditions and investigate its potential mechanisms. To achieve this, a comprehensive approach integrating bioinformatics analyses with both in vitro and in vivo experiments was employed.

Initially, network pharmacology was employed to identify potential targets. By intersecting the effective targets of Rg1 (187) with disease-related genes (606), 46 common targets were discovered. Notably, key proteins such as TNF, IL6, STAT3, AKT1, and TP53 were found to play critical roles in regulating cell survival, inflammation, the cell cycle, and apoptosis. This suggests that Rg1 may exert its protective effects through multiple pathways. Enrichment analysis further supports this hypothesis by highlighting Rg1’s involvement in various biological processes, such as adhesion and regulation, as well as biological functions like antioxidant activity and ion channel regulation. It also impacts cellular components, including cell junctions. These findings suggest that Rg1 enhances intercellular communication and stability, which is crucial for repairing and maintaining lung tissue under hypoxic conditions. The core pathways identified include the fluid shear stress and atherosclerosis pathway, the HIF-1 signaling pathway, and the PI3K-AKT signaling pathway [20,21]. These pathways are closely associated with oxidative stress, vascular function, and cell survival/proliferation [22,23]. Thus, it is plausible that Rg1 may mitigate lung injury induced by high-altitude hypoxia through various signaling networks.

In vivo experiments demonstrated that Rg1 treatment significantly increased the partial pressure and saturation of arterial oxygen in rats, indicating improved oxygen transport and utilization. These findings are consistent with previous studies [24] and suggest that Rg1 plays a crucial role in ameliorating the blood acid-base balance under hypoxic conditions, as evidenced by the significant enhancement of parameters associated with respiratory alkalosis, such as CO_2_ partial pressure, HCO_3_^−^ concentration, BB, and BE. Histological and ultrastructural analysis in vivo showed that Rg1 effectively protected the lung tissue structure by alleviating the thickening of pulmonary microvascular walls, reducing lung tissue injury and immune infiltration, and decreasing the lung index and SP-D protein level. Moreover, Rg1 treatment significantly improved the structure and morphology of mitochondria and endoplasmic reticulum in alveolar epithelial cells. These findings suggest that Rg1 could effectively improve ALI after hypoxia and maintain normal physiological lung function, which is consistent with existing literature. When a pulmonary or extrapulmonary insult occurs, it can lead to the release of inflammatory mediators. These mediators promote the accumulation of inflammatory cells in the alveoli and microcirculation of the lung. As a result, the vascular endothelium and alveolar epithelium are damaged, leading to various consequences such as pulmonary edema, the formation of hyaline membranes, decreased lung compliance, and impaired gas exchange [25]. In vitro experiments further confirmed the protective effects of Rg1. Rg1 treatment significantly improved the survival rate of HPMEC and A549 cells under hypoxic conditions. It also reduced intracellular ROS and Ca^2+^ levels, indicating its ability to alleviate cell damage caused by oxidative stress and calcium imbalance. Mitochondrial dysfunction is known to contribute to hypoxia-induced cell damage. Our results showed that Rg1 treatment enhanced mitochondrial function, particularly in terms of basal respiration and proton leakage, which are crucial for sustaining energy metabolism and normal cellular function. These findings align with previous studies [26] highlighting the importance of protein synthesis, energy metabolism, and mitochondrial respiration in the cellular stress response to hypoxia. Furthermore, in vivo experiments revealed that Rg1 treatment elevated arterial blood glucose levels, suggesting its potential to augment energy supply and alleviate hypoxia.

Hemorheology is known to reflect shear force and microcirculation health [27], with blood viscosity playing a significant role in influencing shear stress [28]. Our results demonstrate that Rg1 effectively reduced compensatory increases in RBCs, HGB, and hematocrit, while ameliorating platelet abnormalities and improving coagulation and hemorheology parameters. These findings suggest that Rg1 mitigates erythrocytosis, platelet deformation, and adhesion induced by hypoxia, restoring coagulation function and reducing blood viscosity, ultimately enhancing hemorheology. The observed improvement in hemorheology can be attributed to the fluid shear stress pathway, which encompasses the forces exerted by blood flow on the vascular wall and blood cells [29]. Moreover, Rg1 enhances the morphology and function of RBCs and platelets, thereby reducing blood viscosity and maintaining normal blood flow and endothelial function. This indicates the role of Rg1 in maintaining hemodynamic stability and enhancing vascular endothelial stability under hypoxic conditions. These effects may be mediated through the regulation of the fluid shear stress pathway, which is crucial for vascular integrity and function during hypoxic stress. Additionally, Rg1 plays a vital role in regulating inflammation. Previous studies have demonstrated that acute hypoxia can lead to lung injury through inflammation and coagulation dysfunction [30]. TNF-α and IL-6 are core cytokines involved in the inflammatory response [31,32]. Our results show that Rg1 reduces the levels of inflammatory cells and cytokines (TNF-α and IL-6) in both blood and alveolar lavage fluid, indicating its anti-inflammatory properties. These effects suggest that Rg1’s anti-inflammatory effect may be mediated through the fluid shear stress pathway, the atherosclerosis pathway, and the PI3K-AKT signaling pathway.

While these findings are promising, this study has several limitations. Firstly, further clarification is required regarding the specific molecular mechanism through which Rg1 regulates the identified targets and pathways. Secondly, additional studies are needed to investigate the dose–response relationship and long-term effects of Rg1. Lastly, although in vitro and in vivo models offer valuable insights, clinical trials are necessary to confirm the therapeutic potential of Rg1 in a plateau environment. Future research directions should include a detailed examination of the molecular interaction between Rg1 and its core target within the context of the fluid shear stress pathway, as well as the determination of the optimal concentration and frequency of Rg1 administration to achieve maximum therapeutic effects. Furthermore, miRNAs and EVs have indeed been implicated in various types of ALI [33]. These small non-coding RNA molecules, specifically miRNAs, have been shown to play crucial roles in the regulation of gene expression and can modulate key signaling pathways involved in the pathogenesis of ALI. In addition, EVs, which include exosomes and microvesicles, have emerged as important mediators of intercellular communication and have been implicated in the transfer of miRNAs between cells. Further studies are needed to fully elucidate the specific mechanisms by which miRNAs and EVs contribute to the processes we investigated.

## 4. Materials and Methods

### 4.1. Materials

Ginsenoside Rg1 (purity 98%, Yuanye Bio-Technology Co., Ltd., Shanghai, China, CAT: B21057-1g); rat interleukin (IL)-6 (CAT: MM-0190R2), tumor necrosis factor (TNF)-α (CAT: MM-0180R2), and surfactant protein-D (SP-D) (CAT: MM-71425R2) enzyme-linked immunosorbent assay (ELISA) kits (Meimian Industrial Co., Ltd., Yancheng, China); sodium pentobarbital (Institute of Chemical Reagents Co., Ltd., Beijing, China, CAT: AM00469); DMEM medium (Gibco Life Technologies, GrandIsland, NY, USA, CAT: C119955500BT); fetal bovine serum (Tianhang Biotechnology Co., Ltd., Shanghai, China, CAT: 13011-8611); penicillin/streptomycin/gentamycin triple antibiotics (Servicebio Technology Co., Ltd., Wuhan, China, CAT: G4014-100ML); trypsin (Sigma-Aldrich, St. Louis, MO, USA, CAT: T4049-100ML); cell counting kit-8 (CCK8, Topscience Co., Ltd., Shanghai, China, CAT: C0005); ROS detection kit (Beyotime Biotech Inc., Shanghai, China, CAT: S0033M); Fluo 3-AM special packaging (Dojindo Laboratories, Kumamoto County, Japan, CAT: F026); mitochondrial oxidative phosphorylation detection kit (Agilent Technologies Inc., Santa Clara, CA, USA, CAT: AIS22013); Hank’s balanced salt solution (HBSS, Biosharp, Hefei, China, CAT: BL559A).

### 4.2. Animals and Grouping

**Figure 10:**
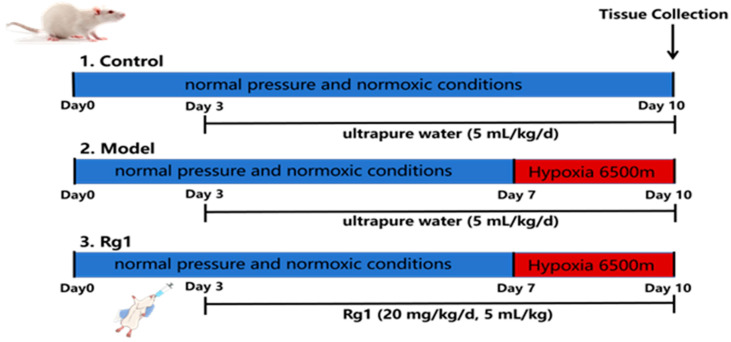
Schematic of the experimental procedure for rats.

Twenty-four male specific pathogen-free (SPF)-grade Sprague Dawley (SD) rats, weighing 180–220 g, were purchased from Beijing Vital River Laboratory Animal Technology Co., Ltd., Beijing, China, with the animal production certificate number SCXK (Jing) 2021-0006. The rats were housed in an SPF-grade animal facility located at the Animal Center of the Academy of Military Medical Sciences. They were maintained under strictly controlled conditions, including a temperature of 26 ± 0.5 °C, relative humidity of 50% ± 5%, and a 12 h light/dark cycle. Each room was equipped with an independent air purification system to maintain optimal air quality. The rats had ad libitum access to sterile distilled water and SPF-grade maintenance feed, and corncob bedding was provided for their comfort. After a 3-day acclimatization period, the rats were randomly divided into three groups: the Control group, the Model group, and the Rg1 group. The Control group was maintained under normobaric normoxic conditions for 10 days. The Model and Rg1 groups were kept under the same conditions for 7 days before being transferred to a multi-factorial environmental simulation medical science cabin (model: DYC-3285, Aviation Industry Corporation of China, Guizhou Fenglei Aviation Ordnance Co., Ltd., Anshun, China) and exposed to a simulated high-altitude environment of 6500 m for 3 consecutive days. Three days after grouping, the Rg1 group received intragastric administration of Rg1 at a dosage of 20 mg/kg/day with an administration volume of 5 mL/kg for 7 days. The Control and Model groups were given an equivalent volume of ultrapure water (Figure 10).

All animal experiments were conducted in accordance with the guidelines of the European Community and were approved by the Institutional Animal Care and Use Committee of the Academy of Military Medical Sciences (IACUC-AMMS), ethical review number IACUC-DWZX-2024-588.

### 4.3. Network Pharmacology

Rg1 was searched in the TCMSP [34] (Traditional Chinese Medicine Systems Pharmacology Database and Analysis Platform, https://old.tcmsp-e.com/tcmsp.php, accessed on 20 May 2024). Potential targets of Rg1 were predicted using the PubChem [35] (https://pubchem.ncbi.nlm.nih.gov/search, accessed on 20 May 2024) and SwissTargetPrediction [36] (http://swisstargetprediction.ch/, accessed on 20 May 2024) databases, considering targets with a Probability > 0 as effective. The terms “acute lung injury” and “high altitude” were used to search for disease-related genes in the DrugBank [37] (https://go.drugbank.com, accessed on 20 May 2024), OMIM (Online Mendelian Inheritance in Man, https://www.omim.org, accessed on 20 May 2024), GeneCards [38] (https://www.genecards.org, accessed on 20 May 2024), TTD [39] (Therapeutic Target Database, http://db.idrblab.net, accessed on 20 May 2024), and PGKB [40] (PharmGKB, https://www.pharmgkb.org, accessed on 20 May 2024) databases. Venn diagrams were constructed using the Microbiosinfo website to identify intersection genes [41]. These intersection genes were imported into String 11.5 (https://string-db.org, accessed on 20 May 2024) to construct the protein–protein interaction (PPI) network, followed by topological analysis using Cytoscape 3.10.0 software. For Kyoto Encyclopedia of Genes and Genomes (KEGG) and Gene Ontology (GO) enrichment analysis, the genes were imported into the DAVID database [42,43] (https://david.ncifcrf.gov, accessed on 20 May 2024).

### 4.4. Blood Gas Analyses

Following 72 h of hypoxia, the rats were euthanized with pentobarbital sodium serving as the anesthetic agent. The abdominal aorta was surgically exposed, and blood was drawn using anticoagulant syringes preloaded with 3% heparin sodium (0.05 mL per syringe). The collected samples were then transferred into a blood gas card for analysis with a blood gas biochemical analyzer (BGA-102, Wondfo Biotech Co., Ltd., Guangzhou, China). Measurements were taken of the partial pressures of gasses such as O_2_ and CO_2_ dissolved in the blood from the abdominal aorta, as well as parameters pertaining to acid-base balance.

### 4.5. Peripheral Blood Cell Counts and Hemorheology Examination

Blood was drawn from the rat’s abdominal aorta using an arterial blood needle and collected into heparin sodium vacuum anticoagulant tubes. A blood cell analyzer (XN-1000V, Sysmex Corporation, Kobe, Japan) was used to perform peripheral blood cell counts, including the quantification of white blood cells (WBCs), red blood cells (RBCs), hemoglobin (HGB), and other parameters. Additionally, an automatic hemorheology analyzer (ZL-6000, ZHONGCHI WEIYE Technology Development Co., Ltd., Beijing, China) was employed to measure blood viscosity and erythrocyte deformability.

### 4.6. Coagulation Profile Testing

Blood was drawn from the rat’s abdominal aorta using an arterial blood needle and collected into sodium citrate vacuum anticoagulant tubes (1:9). The plasma was then tested to determine the prothrombin time (PT), activated partial thromboplastin time (APTT), thrombin time (TT), and fibrinogen (FIB) levels.

### 4.7. Inflammatory Response Assessments

The trachea was dissected, intubated, and lavaged three times with 1 mL of pre-chilled sterile saline at 4 °C, yielding a total volume of 3 mL. The bronchoalveolar lavage fluid (BALF) was collected in sterile centrifuge tubes, and the supernatant was obtained after centrifugation. Blood was drawn from the rats’ abdominal aorta using an arterial blood needle and collected into vacuum tubes without any anticoagulant. After resting for 2 h, the blood was centrifuged to obtain serum. ELISA kits were used to measure the levels of IL-6 and TNF-α in both BALF and serum.

For lung tissue analysis, precisely 20 mg of lung tissue from a group of three rats was weighed and homogenized with 180 µL of pre-chilled PBS. The supernatant was collected after centrifugation and analyzed for surfactant protein D content using the respective ELISA kits.

All procedures were performed according to the manufacturers’ instructions, which included steps such as sample addition, enzyme addition, incubation, washing, color development, and termination. Finally, the absorbance of each well was measured sequentially at a wavelength of 450 nm.

### 4.8. Calculation of Lung Index

Each rat was weighed prior to euthanasia, after which the lung tissue was extracted and weighed to calculate the organ index. The lung index of a specific rat from the Control group was designated as 1, serving as a reference to determine the relative lung indices of the other rats.

Lung Index = Lung Weight (mg)/Body Weight (g)

Relative Lung Index = Lung Index of each rat/Lung Index of the Control group rat

### 4.9. Histopathological Analysis

Hematoxylin and Eosin (H and E) Staining: Lung tissues were harvested from groups of three rats and fixed in 4% paraformaldehyde to preserve their structural integrity. The tissues were subsequently dehydrated, embedded in paraffin, and sectioned into slices of approximately 5 µm in thickness. The sections underwent hematoxylin staining for 10 to 15 min, and then were rinsed in distilled water. Eosin staining was then applied for 15 s, succeeded by an additional rinse with distilled water. The sections were dehydrated through an ethanol gradient of 75%, 85%, 95%, and finally 100%, with each step lasting 2 min. This process was followed by clearing in xylene and mounting with neutral resin. Pulmonary microvessels with diameters less than 300 μm were selected for analysis. The wall thickness of these microvessels was determined using CaseViewer software (2.4.0). The relative thickness of the pulmonary microvascular wall was calculated as the ratio of the wall thickness to the microvascular radius.

Transmission electron microscopy (TEM) observation: The rat lung tissues were pre-fixed with glutaraldehyde, washed with phosphate-buffered solution, and post-fixed with osmium tetroxide. They then underwent dehydration, infiltration, embedding, and sectioning. The sections were stained with heavy metals to examine the morphological structure of alveolar epithelial cells and subcellular structures such as mitochondria and rough endoplasmic reticulum.

### 4.10. Construction of Hypoxia Cell Model

HPMEC and A549 cells were exposed to hypoxic conditions of 1% O_2_ and 5% CO_2_ for periods of 12, 24, and 48 h, respectively. The growth state and cell density were observed under a microscope, and cell viability was assessed using a CCK8 assay kit.

### 4.11. Detection of Cellular ROS Release and Ca^2+^ Influx

ROS Detection: After a single wash with pre-cooled PBS, the cells were digested with EDTA-free trypsin and collected. They were then washed once more with PBS. A diluted dye was added, and the cells were incubated for 20 min in a 37 °C cell culture incubator to ensure full interaction between the probe and the cells. Following incubation, the cells were washed three times with a serum-free culture medium. Detection was performed at an excitation wavelength of 488 nm and an emission wavelength of 525 nm.

Ca^2+^ Detection: A working solution of Fluo 3-AM at a concentration of 2 µmol/L was prepared. The cells were collected and washed once with HBSS. The working solution was added, and the cells were incubated for 30 min in a 37 °C cell culture incubator. After incubation, the cells were washed three times with HBSS. Detection was performed using an excitation wavelength of 480–500 nm and an emission wavelength of 525–530 nm (FITC channel).

### 4.12. Detection of Cellular Mitochondrial Respiratory Capacity

Cells were seeded into a Seahorse XF cell culture plate and incubated overnight prior to the experiment. The Seahorse XFe96 Analyzer (Agilent, Santa Clara, CA, USA) along with its accompanying computer and software were activated to stabilize at 37 °C, allowing for overnight preheating. Probes were hydrated in a CO_2_-free incubator at 37 °C throughout the night. On the day of the experiment, the following procedures were conducted: the assay medium was prepared; cell status was assessed; the growth medium was replaced with the assay medium, followed by incubation of the cells in a CO_2_-free incubator at 37 °C for 1 h; reagents were prepared and added accordingly; experimental conditions were documented and the assay program was designed using the software; finally, the assay was executed. Subsequently, the experimental data were analyzed utilizing Wave software (2.6) and the Report Generator.

### 4.13. Statistical Analyses

In this experiment, all data were obtained from more than three independent replicates and are expressed as the mean ± standard deviation. GraphPad software (9.0) was utilized for data visualization. Statistical analysis among multiple groups was performed using one-way analysis of variance (ANOVA), followed by post hoc tests for pairwise comparisons. Comparisons between two groups were conducted using the *t*-test. A *p*-value of less than 0.05 was considered statistically significant.

## 5. Conclusions

This study highlights the substantial protective effects of Rg1 in mitigating ALI induced by high-altitude hypoxia. Through a combination of in vivo and in vitro experiments, it was demonstrated that Rg1 enhances oxygen delivery and utilization, diminishes oxidative stress and inflammatory responses, and preserves cellular metabolism and vascular function. Bioinformatics analysis indicates that Rg1 may exert its protective effects through various signaling pathways, including the shear stress pathway and the PI3K-AKT signaling pathway.

## Data Availability

Data will be made available on request.

## Abbreviations

ALI	Acute lung injury
AMS	Acute mountain sickness
APTT	Activated partial thromboplastin time
BALF	Bronchoalveolar lavage fluid
BB	Buffer base
BE	Base excess
SpO_2_	Blood oxygen saturation
HCO3^−^	Bicarbonate
BP	Biological processes
CC	Cellular components
CCN1	Cellular communication network factor 1
CCK8	Cell counting kit-8
ECs	Endothelial cells
ELISA	Enzyme-linked immunosorbent assay
FIB	Fibrinogen
GO	Gene Ontology
HAPE	High-altitude pulmonary edema
HBSS	Hank’s balanced salt solution
HCT	Hematocrit
H&E	Hematoxylin and Eosin
HGB	Hemoglobin
HPH	Hypoxic pulmonary hypertension
HPMEC	Human pulmonary microvascular endothelial cells
HIF	Hypoxia-inducible factors
IL	Interleukin
KEGG	Kyoto Encyclopedia of Genes and Genomes
LSS	Laminar shear stress
LYMPH	Lymphocytes
MFs	Molecular functions
MONOs	Monocytes
MPV	Mean platelet volume
NEUTs	Neutrophils
OCR	Oxygen consumption rate
OMIM	Online Mendelian Inheritance in Man
OSS	Oscillatory shear stress
PaCO_2_	Partial pressure of arterial carbon dioxide
PaO_2_	Partial pressure of arterial oxygen
PDE5	Phosphodiesterase type 5
PDW	Platelet distribution width
PGKB	PharmGKB
PI3K	Phosphoinositide 3-kinase
P-LCR	Platelet–larger cell ratio
AKT	Protein kinase B
PPI	Protein–protein interaction
PT	Prothrombin time
RBCs	Red blood cells
ROS	Reactive oxygen species
SD	Sprague Dawley
SP-D	Surfactant protein-D
SPF	Specific pathogen-free
TEM	Transmission electron microscopy
TT	Thrombin time
TTD	Therapeutic Target Database
TCM	Traditional Chinese medicines
TCMSP	Traditional Chinese Medicine Systems Pharmacology Database and Analysis Platform
TNF	Tumor necrosis factor
WBCs	White blood cells
